# Optimizing expertise management in crowdsourcing contests: The impact of information structures on open innovation efficiency

**DOI:** 10.1016/j.fmre.2024.10.011

**Published:** 2024-10-28

**Authors:** Lei Lin, Xupeng Wang, Hongbo He, Yihua Du, Runqiang Wang, Li Xu

**Affiliations:** a Computer Network Information Center, Chinese Academy of sciences, Beijing 100083 China; b Institute of Computing Technology, Chinese Academy of sciences, Beijing 101408 China

**Keywords:** Crowdsourcing contest, Innovation contest design, Mechanism design, Mixed-strategy equilibrium, Lying flat, Involution

## Abstract

For companies and governments alike, crowdsourcing contests have emerged as a popular approach to harness the benefits of open innovation. Consequently, numerous game models have been developed to analyze these contests and model the interactions in equilibrium between a contest's sponsors and its participants. Some models assume incomplete information where player abilities are private information, while others model complete information scenarios where these abilities are revealed. However, to date, no uniform framework has been proposed to comparatively analyze the theoretical outcomes of these two distinct information structures. This paper presents a formulation to derive a complete information model from a previously published incomplete information model using the variational method. This formulation allowed us to derive the effort levels of participants given a mixed-strategy equilibrium in a single-prize, complete information crowdsourcing contest. We then conducted a comparative analysis between the complete and incomplete information models, considering contests with and without a registration phase (i.e., where the sponsor knows/does not know the participants' abilities beforehand). The key findings are as follows. With a registration phase, incomplete information tends to yield higher quality solutions when the abilities of top participants exceed a certain skill threshold. Without registration, complete information may lead to better solutions, especially when the sponsors have a tendency toward risk-taking. Finally, the complete information mixed-strategy equilibrium is used to provide a game-theoretic interpretation of the recent 'involution' and 'lying flat' phenomenon observed in China, where players withdraw from a contest when competition is excessive. This offers the first explanation for this behavior based on an equilibrium strategy, in contrast to the previous explanations, which are all psychological.

## Graphical abstract

**Figure:**
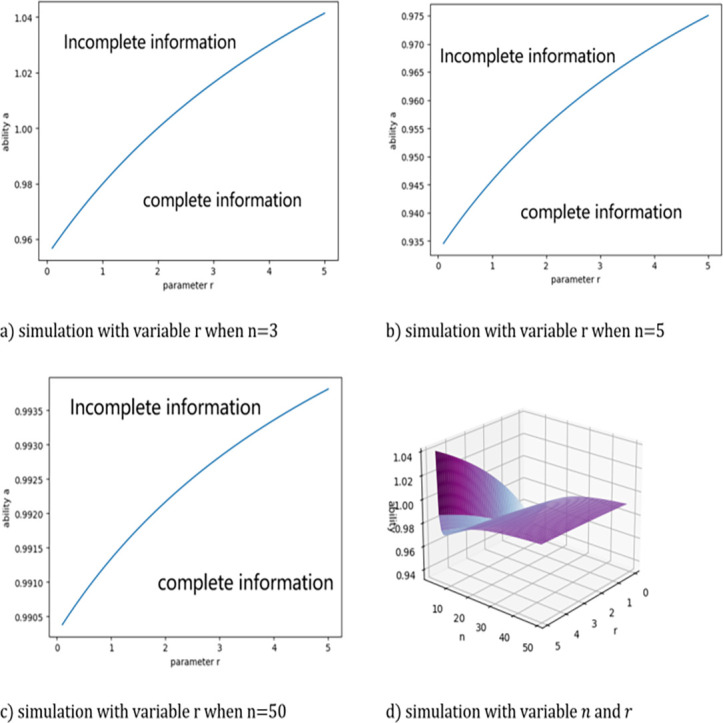

## 1 Introduction

Over the last two decades, open innovation has become a popular business model, with more and more organizations adopting this concept to accelerate their technical innovations [1]. Further, both large corporations and SMEs alike [[2], [3], [4], [5]] are implementing open innovation as a valid strategy for rapidly commercializing their emerging products and augmenting their internal innovation teams.

It is in the very nature of open innovation to see knowledge flowing across the boundaries of organizations [6]. As Prof. Chesbrough, the father of open innovation, states “the use of purposive inflows and outflows of knowledge to accelerate internal innovation, and expand the markets for [its] external use” makes knowledge flows one of the most fundamental aspects of open innovation [1]. Accordingly, open innovation is often divided into two types: inbound and outbound open innovation. Outbound open innovation, or exports of knowledge, exploit the knowledge within a firm's boundaries, using it to discover fruitful collaborations and new market opportunities. This knowledge typically takes the form of inventions, techniques, solutions, etc. Furthermore, outbound open innovation is generally very good at overcoming bottlenecks in innovation because it not only solves problems but also has the tendency to transform ecosystems at the same time [7].

Inbound open innovation, or imports of knowledge, has attracted much scholarly attention [1,6,8]. This type of open innovation looks for sources of knowledge outside a firm to develop technologies or products through collaborations with other organizations or experts. This is the category crowdsourcing contests fall into. Such contests are a powerful and robust way to approach open innovation. As an example, Netflix [9] published an open call for developers in the data and computer science communities to improve the RMSE of their movie ratings regression algorithm by 10%. The prize for the best solution was $1 million. As crowdsourcing contests have grown in popularity, several online platforms that facilitate such competitions have also come into being, including InnoCentive.com [10], Kaggle [11], Climate Colab [12], and TopCoder [13]. All of these platforms facilitate the process of announcing calls, submitting solutions, evaluating those solutions, and awarding prizes, where all contestants compete against each other for one or more rewards.

These contests comprise two types of stakeholders: the contest sponsor and the participants. The sponsor creates an open call to solve a particular problem for a certain reward, generally using an online contest platform. The participants are the problem solvers who invest their efforts into finding a solution for the published problem, either for intrinsic reasons, like altruism or pure enjoyment, or for extrinsic rewards, such as money. Notably, both types of incentives play an important role in these contests [14]. For the participants, equilibrium is a function of the effort put into finding a solution balanced with the reward obtained for doing so. For the sponsor, equilibrium means designing a mechanism that provides sufficient information and rewards to incentivize the participants to contribute workable, feasible solutions, but does not over or undershoot the desired quality.

More specifically, when sponsors decide to use crowdsourcing contests to solicit solutions, they generally have two concerns. One is the reward distribution, i.e., how many prizes will be provided to which participants and the total sum of rewards given out as part of the contest. The other is the private information to be disclosed, such as the participants’ names, which will obviously give away the different abilities of the competitors. The sponsor's objective is to balance the monetary rewards offered with the quality of solicited solutions. From the participant's perspective, each contestant must balance the effort they expend in finding the solution with the rewards offered. Here, they have two decisions to make. The first is whether to participate in the contest at all. The second concerns how much effort they will put into winning. Naturally, the more effort they exert, the better the quality of the solution they will provide to sponsors, and the more chance they have to win the final prize. Their objective is therefore to maximize their monetary award.

Another important aspect of these contests to consider is that they are often staged to improve efficiency. In such formats, the participants are iteratively eliminated through multiple rounds. As examples, Climate Colab [12] separates the contestants into semi-finalists and finalists, while PRESANS [15] uses “Multistep Dynamic Expert Sourcing” to select experts through three different stages of filtering. Mechanisms like this type of short listing are also popular in areas like human resources, R&D races, and sports. The advantage of using some type of hierarchical selection mechanism is that it can help the sponsor to find a small group of participants with particularly strong abilities in the problem domain. Thus, the sponsor can incentivize this smaller group to exert more effort for higher quality solutions [16]. It therefore follows that information on the participants’ abilities should be vital to the contest sponsors, as they will tend to want to collaborate with the participants who have the deepest knowledge and experience with the problem at hand.

Another thing that crowdsourcing contest platforms generally try to do is to build a community around solving a problem. Often, there is even a point system to track and record each participant's contributions to the contest. Platforms like Crowdsprint, 99designs, Climate Colab [12], and Kaggle even allow the contestants to check each other's historical work and performance. Further, in data science contests, like Netflix [9] and Kaggle [11], the participants are able to see the current scores of each player and their ranking on a leaderboard. Intuitively, this kind of information disclosure strategy should give rise to a tough competition that benefits the sponsor, especially when participants with very strong abilities are involved.

However, other open innovation platforms keep the participants’ information private. For example, the well-known and pioneering platform InnoCentive.com [10] offers open calls for innovation challenges but ensures that each contestant's information stays invisible [17]. The challenge solvers are not only unaware of who they are competing with, but also how many solutions have been submitted. Interestingly, this platform has achieved great success with challenges involving chemistry, the physical sciences, engineering/design, mathemtics, and other similar fields [10].

Intrigued by the difference in information disclosure of the various platforms, we decided to investigate the influence that knowing or not knowing a competitor's abilities made to the contest outcome. To the best of our knowledge, no study has yet compared the performance of a complete information model versus an incomplete information model in the context of a crowdsourcing contest. Some studies have analyzed this juxtaposition in an all-pay auction model with complete information [16,18,19], in which the abilities and rewards are not separate and not easily amenable to analysis [20]. The recent literature also considers private-information contests that involve separate ability information and cost functions reflecting marginal effects, such as all-pay contests with incomplete information. This type of incomplete information model is ideal for studying the efficient allocation of rewards in a contest [16,[20], [21], [22], [23]]. However, the main question to be addressed with this paper is to compare the equilibrium points of two contest models – one with complete information and the other with incomplete information – so as to help contest sponsors determine whether or not disclosing ability information will produce better outcomes from the competition.

To answer this question, we theoretically analyzed a winner-take-all crowdsourcing contest, in which the sponsor announces an open call with a single final reward, then the competitors decide whether to participate. This contest structure, taken from [21], is then modelled with both incomplete information, as is the case in [21], and with complete information by making the contestants’ ability information visible.

There are three main contributions of this work. First, the paper presents a mixed-strategy equilibrium problem posed as a variational problem with definitions and solutions based on a variational approach. Second, the theoretical proofs provide a counterintuitive economic conclusion resulting from the convex marginal cost effort under competition. The results demonstrate that, when the top players decide to participate, an incomplete information contest is better than a complete information contest, especially for risk-neutral and risk-averse sponsors. Third, we examine the concepts of ‘involution’ and ‘lying flat’ recently popularized in China, which refers to the phenomenon of players withdrawing from a contest due to excessive competition. Where most researchers have interpreted this behavior through the lense of social or pyschological constructs, we draw a link between this behavior and a microeconomic equilibrium strategy.

The rest of this paper is organized as follows. Section 2 summarizes the work most closely related to this research. Section 3 presents the models and solutions and derives the equilibrium of the complete information scenario. Section 4 derives the equilibrium for the incomplete information scenario and compares the results to the complete scenario. Section 5 discusses the theoretical and practical implications of these proofs for management and economics, followed by some concluding comments in Section 6.

## 2 Related work

Crowdsourcing contests involve multiple parameters that can affect the competition's outcomes. For example, some contests involve multiple prizes, such as Climate Colab [12], where two prizes are generally offered for the top two proposals. Some contests comprise multiple stages, such as the Multistep Dynamic Expert Sourcing framework [15], which filters the most relevant experts for a task through three different stages. Further, some contests make player information, including their names and prior achievements, visible, as is the case with Kaggle's data science competitions [11], while others hide such information. This study specifically aims to compare the impact of information disclosure as a parameter in crowdsourcing contests. In other words, the goal is to compare maximum utility and contest outcomes for the sponsor given a complete information model versus an incomplete information model.

The literature on estimating crowdsourcing contest models begins with all-pay auction models [18,24,25]. The first of these studies simply modelled these contests as all-pay auctions, where each participant paid an amount to compete no matter whether they “won” or not – the amounts paid being proxies for the effort expended. These models involved maximum bounds on the amounts, which depended on private utility information. For example, Hillman and Riley [24] were the first to compare equilibrium behavior in all-pay auctions given complete and incomplete information models. They reached the conclusion that the participant who expended the most effort would win the prize by making a maximum bid under incomplete information. By contrast, the participant with the highest skills unexpectedly won the prize with a mixed-strategy under complete information. Further correcting Hillman and Riley's proof [24], Baye et al. [18] completely characterized the equilibrium behavior found in a complete information all-pay auctions with single prize. That said, contests with multiple prizes are quite common in the real world – take the Olympic Games as an example with its gold, silver, and bronze medals. So, to explore whether multiple prizes might prompt participants to exert more or less effort, Clark and Christian [26] analyzed the contest models involving multiple prizes – again based on all-pay auction models, but this time with complete information. In addition, Barut and Kovenock [26] generalized the prize parameters, proposing an all-pay model with multiple identical zero rewards. This had the effect of unifying previous models such that the single prize model became a special case of multiple prize models. However, although an all-pay auction model can characterize each of the contestant's contributions as one valuation parameter, such models are not particularly suited to analyzing the marginal cost behavior of the contestants [20].

In many ways, open innovation contests are not like auctions. For example, in a contest, the contestants’ abilities should be defined as a value parameter, and this value should determine the cost of a given solution, i.e., the greater the ability, the less it should cost to develop a solution of the same quality. Therefore, to investigate the marginal cost behavior of different contestants, Moldovanu and Sela [20] designed separate ability and effort parameters to describe the costs in each contestant's utility function. They then analyzed linear, convex, and concave cost functions with multiple prizes. Overall, they concluded that offering multiple prizes was better than offering a single prize with a convex cost function, and vice versa for linear and concave cost functions. However, Cheng et al. [21] and Gou [27] both reached the opposite conclusion for convex cost functions. This is because both these teams maximized the expected greatest effort by contestants as the sponsor utility instead of maximizing the expected total effort expended by contestants.

Terwiesch and Xu [28] uniquely devised three variables to model the quality of solutions: private variable expertise β, effort e, and random noise ξ. Here, ξ measures the level of uncertainty in the contest, while β is a threshold for highly specialized work. They conclude that more contestants entering the contest will reduce the effort expended and also lower the probability of winning given an incomplete information scenario. Körpeoğlu and Cho [29] further generalized the models in [20,21,27,28] by introducing a productivity parameter a, which models ability as the coefficient of effort e in a quality function. The conclusions reached are consistent with [21,27], but run counter to the conclusions in [28], which is that increasing the number of contestants reduces the effort required to reach equilibrium. Rather than using a convex cost function of effort, as is the case in [20,21,27], some studies rely on a concave function to model marginal behavior, e.g., [28,29]. Notably, however, all these models involve incomplete information. They also focus on the utility function for the contestants and sponsors as well as on the reward allocations. What they do not consider is the role that complete information plays in reaching equilibrium.

With the development of one grand static contest model, scholarly attention turned to improving contest architectures. For example, Moldovanu and Sela [23] furthered their studies by modelling parallel sub-contests, comparing the results with a single-prize contest and different payoffs by the sponsor. Their results show that parallel sub-contests can be beneficial from the standpoint of the convex cost function if the payoff function is the expected total effort. Obviously, it is understood that, with a convex cost function, the prizes are split between more contestants who pursue more beneficial marginal costs. Cohen et al. [30] modelled a two-stage elimination Tullock contest with an optimal head start, which yielded a higher expected total effort than one grand contest. In fact, contests with two or more stages typically have two functions. One function is to filter out contestants with lower abilities. This is of benefit when more participants entering the contest will reduce the overall effort required to win. The other function is to reveal the contestants’ private information, i.e., their abilities, in the first stage of the contest. Hou and Zhang [16] studied two types of two-stage contest – these being sequential elimination [30] and sub-elimination [23]. They find that having a small number of contestants in the second stage yields the most benefit, and that sequential elimination is a more optimal format than one-stage or sub-elimination contests. In addition, sequential-elimination is also of benefit because the contestants can update their beliefs on the abilities of others in the second stage simply from observing the results of the first stage. Notably, Jiang and Wang [31] modelled the idea of the sponsor providing feedback during the contest by subtly exploiting the property of Gaussian posterior distribution. In this model, although the sponsor provides feedback to the contestants in the form of comments, this information is not made available to the other players. Therefore, the sponsor benefits but the contestants are not necessarily incentivized to expend more effort. These studies not only indicate that the sponsor can benefit from revealing information, but also that information disclosure plays an important role in incentivizing contestants.

This study differs from previous research on contest modeling in several respects. First, this is the first study to compare a complete information contest with an incomplete information contest in different scenarios. The model presented is closely related to [21] except that ability information about the contestants is revealed at the beginning of the contest, which intuitively prompts higher-ability contestants to put more effort into the competition. Because there is no pure equilibrium strategy in a complete information contest, comparisons with incomplete information are made using probability metrics. Second, this research also differs from those studies who have used an auction model as a base [24] in that the contestants’ marginal cost behaviors are analyzed with different sponsor payoff functions. Third, the complete information contest model provides an explanation for competitive economic phenomena.

## 3 Model development

This section of the paper considers a crowdsourcing contest with two groups of stakeholders: the sponsor and the participants. The sponsor publishes an open call for solutions to his problem, and the participants provide solutions to the sponsor. After evaluating the participants’ solutions, the sponsor selects a winner who will receive a monetary reward. The remaining participants will receive nothing. This contest format is often called a winner-take-all structure.

The model to be presented only considers the characteristics of the participants and the sponsor. The quality of the solution is measured in one dimensional space. Formally, the quality of solution i submitted by participant i (i=1,⋯,n) is denoted by the real quantity $q_{i}$. Additionally, in this winner-take-all structure, the sponsor only pays a reward R to one final winner. It is assumed that the utility function of the sponsor is $U\left(q\right)=q^{r}$, in which q≥0 is the quality of the solution and r>0 is the risk parameter – this being a scale from risk-averse to risk-loving [32]. This parameter is useful for comparing the expected utility maximization under different distributions of q. Obviously, if 1>r>0, the sponsor is risk averse; if r=1, the sponsor is risk neutral; and if r>1, the sponsor is a risk lover. The sponsor's payoff from the contest will be

(1) $$\Pi =\max_{i}U\left(q_{i}\right)=\max_{i}q_{i}^{r}$$

Commonly, it is assumed that the quality $q_{i}\;$of submission i is equal to the effort $e_{i}$ expended by participant i, i.e., $q_{i}=e_{i}\;$.

Second, the participants are assumed to differ in terms of their abilities $a_{i}$, which describes their expertise at developing a potential solution. Given a solution of the same quality q, a participant with greater ability should exert less effort, i.e., less cost, to create a solution. Thus, the cost function for the participants is defined as C(a,x), which monotonically increases with effort x and monotonically decreases with ability a. Thus, for a given ability a and cost c, the inverse function for cost is denoted as $x=C_{a}^{-1}\left(c\right)$. Obviously, given a cost c, $C_{a}^{-1}\left(c\right)$ increases with ability a. In addition, no effort equals no cost, i.e.,

(2) C(a,x)=0,ifx=0

In a winner-take-all structure, the participant who expends the most effort will generally win the reward R. The utility function $u_{i}$ of a participant i with ability $a_{i}$ is a function of her effort $x_{1},...,x_{n}$. This is calculated as

(3) $$\begin{array}{ccc} & & U_{i}\left(x_{1},...,x_{n}\right)\\ & & =\left\{ \begin{array}{cc} -C\left(a_{i},x_{i}\right) & if\;\exists j\;s.t.\;x_{j}>x_{i}\\ \frac{R}{m}-C\left(a_{i},x_{i}\right) & if\;participant\;i\;exert\;same\;effects\;as\;m-1\;others\\ R-C\left(a_{i},x_{i}\right) & if\;x_{i}>x_{j}\;for\;\forall j\neq i \end{array}\right. \end{array}$$

### 3.1 Equilibrium strategy for complete information

In a complete information game, i.e., where each participant knows each others’ abilities, there is obviously no pure-strategy equilibrium. However, suppose that participant i with ability $a_{i}>0$ reaches a mixed-strategy equilibrium for his effort $x_{i}$, and that the cumulative distribution function over effort is denoted as $F_{i}\left(x\right)$. The relaxed boundary conditions will then be

(4) $$F_{i}\left(0\right)\geq 0,\;F_{i}\left(+\infty \right)=1$$

Futher, let ${\bar{e}}_{i}\geq {\underset{‾}{e}}_{i}\geq 0$ denote the lower and upper bounds of the cumulative distribution function for the effort function $F_{i}\left(x\right)$, and let $P_{i}\left(x\right)$ denote the size of the mass point in $F_{i}\left(x\right)$. The following expressions then formulate the participants’ equilibrium under an expected surplus with $F_{i}\left(x\right)$.

Typically, a participant i will win the final reward if, and only if, the quality $q_{i}$ of her solution is better than the solutions of others. The probability of participant i winning the final reward is therefore

(5) $$P_{Ci}\left(q_{i}\right)=\text{Pr}\left\{q_{i}>max_{j\neq i}\left(q_{j}\right)\right\}$$

According to the previous assumption, the quality $q_{i}\;$of submission i is equal to the effort $e_{i}$ that participant i expends, i.e., $q_{i}=e_{i}\;$. Thus, $P_{Ci}$ formulated by $e_{i}$ is

(6) $$\begin{array}{ccc} P_{Ci}\left(e_{i}\right) & = & \text{Pr}\left\{e_{i}>max_{j\neq i}\left(e_{j}\right)\right\}\\ & = & \prod_{j\neq i}F_{j}\left(e_{i}\right) \end{array}$$

While, the expected cost for participant i is

(7) $$E\left(C\left(a_{i},e\right)\right)=\int C\left(a_{i},x\right)dF_{i}\left(x\right)$$

Thus, participant i’s expected surplus is

(8) $$\begin{array}{ccc} \pi_{i} & = & E\left(U_{i}\right)\\ & = & E\left(R\times P_{Ci}\right)-E\left(C\left(a_{i},e\right)\right)\\ & = & \int_{{\underset{‾}{e}}_{i}}^{{\bar{e}}_{i}}\left(R\prod_{j\neq i}F_{j}\left(x\right)-C\left(a_{i},x\right)\right)dF_{i}\left(x\right) \end{array}$$

In order to further characterize the expected surplus under a mixed-strategy equilibrium $F_{i}\left(x\right)$, we discuss the upper bound and lower bound of effort ${\bar{e}}_{i},{\underset{‾}{e}}_{i}$ in the equilibrium strategy of any participant i, under the assumption that n≥2 and $1\geq a_{1}>a_{2}...>a_{n}>0$.

Lemma 1 *for* $\forall i,\;C_{a_{i}}^{-1}\left(R\right)\geq {\bar{e}}_{i}\geq {\underset{‾}{e}}_{i}\geq 0$.

Proof:

It is obvious that each participant can either be inactive, expend zero effort, or expend less cost than the reward R offered. Q.E.D. □

Lemma 2 *if* ∃i*such that* ${\underset{‾}{e}}_{i}>{\underset{‾}{e}}_{j}$*, then* ${\underset{‾}{e}}_{j}=0$ *and* $F_{j}\left(0\right)=\lim_{x\to {\underset{‾}{e}}_{i}+0}F_{j}\left(x\right)$*. In addition, if* ${\underset{‾}{e}}_{i}>{\underset{‾}{e}}_{j}$ *and* $P_{i}\left({\underset{‾}{e}}_{i}\right)$ *= 0, then* $F_{j}\left(0\right)=F_{j}\left({\underset{‾}{e}}_{i}\right)$ i.e.*,* $P_{j}\left({\underset{‾}{e}}_{i}\right)$ *= 0. Not only that, if* ∃i *such that* ${\underset{‾}{e}}_{i}={\underset{‾}{e}}_{j}$ *and* $P_{i}\left({\underset{‾}{e}}_{i}\right)$ *= 0, then* ${\underset{‾}{e}}_{j}={\underset{‾}{e}}_{i}=0$.

Proof:

Let $u_{j}\left(x_{j},F_{-j}\right)$ denote j's payoff for his effort $x_{j}$ when strategy $F_{-j}$ is employed by the other n-1 participants. Now, if ${\underset{‾}{e}}_{i}>{\underset{‾}{e}}_{j}$ if ${\underset{‾}{e}}_{j}>0$, $u_{j}\left({\underset{‾}{e}}_{j},F_{-j}\right)=-C\left(a_{j},{\underset{‾}{e}}_{j}\right)<0$, which will conflict with the equilibrium strategy. Thus, ${\underset{‾}{e}}_{j}=0$. Obviously, $F_{j}\left(0\right)=\lim_{x\to {\underset{‾}{e}}_{i}+0}F_{j}\left(x\right)$. If not, $\exists \theta \;s.t.\;{\underset{‾}{e}}_{j}=0\leq \theta \leq {\underset{‾}{e}}_{i}$, $F_{j}\left(\theta \right)>F_{j}\left(0\right)$. However, for $\forall e\in \left({\underset{‾}{e}}_{j},\theta \right],\;u_{j}\left(e,F_{-j}\right)=-C\left(a_{j},e\right)<0$, which will conflict with the equilibrium strategy.

In addition, since $F_{j}$ is an increasing function if $F_{j}\left({\underset{‾}{e}}_{i}\right)>F_{j}\left(0\right)$ when ${\underset{‾}{e}}_{i}>{\underset{‾}{e}}_{j}=0$ and $P_{i}\left({\underset{‾}{e}}_{i}\right)$ = 0, then $\exists \theta \in (0,{\underset{‾}{e}}_{i})$ or $P_{j}\left({\underset{‾}{e}}_{i}\right)$ > 0, such that participant j exerts effort toward the equilibrium strategy, i.e., $u_{j}\left(\theta ,F_{-j}\right)=-C\left(a_{j},\theta \right)<0$ or $u_{j}\left({\underset{‾}{e}}_{i},F_{-j}\right)=-C\left(a_{j},{\underset{‾}{e}}_{i}\right)<0$ when $P_{i}\left({\underset{‾}{e}}_{i}\right)$ = 0. This will also conflict with the equilibrium strategy; hence, the claim $F_{j}\left({\underset{‾}{e}}_{i}\right)=F_{j}\left(0\right)$ follows. Obviously, if ${\underset{‾}{e}}_{i}={\underset{‾}{e}}_{j}>0$ and $P_{i}\left({\underset{‾}{e}}_{i}\right)$ = 0, it holds that $u_{j}\left({\underset{‾}{e}}_{j},F_{-j}\right)=-C\left(a_{j},{\underset{‾}{e}}_{j}\right)<0$, which again conflicts with the equilibrium strategy. Thus, ${\underset{‾}{e}}_{j}=0$. Q.E.D. □

Lemma 3 *for* $\forall i,\;{\underset{‾}{e}}_{i}=0.$

Proof:

Without loss of generality, suppose that ${\underset{‾}{e}}_{1}\geq {\underset{‾}{e}}_{2}\geq ...\geq {\underset{‾}{e}}_{n}$. From here, two situations arise: one where ${\underset{‾}{e}}_{1}={\underset{‾}{e}}_{2}=...={\underset{‾}{e}}_{m}>{\underset{‾}{e}}_{m+1}\geq ...\geq {\underset{‾}{e}}_{n}$, and the other where ${\underset{‾}{e}}_{1}>{\underset{‾}{e}}_{2}\geq ...\geq {\underset{‾}{e}}_{n}$. However, it is possible to prove that neither of these two situations exist. Tackling the first situation first, according to Lemma 2, ${\underset{‾}{e}}_{1}>{\underset{‾}{e}}_{m+1}={\underset{‾}{e}}_{m+2}..=\;{\underset{‾}{e}}_{n}=0$. Because ${\underset{‾}{e}}_{1}={\underset{‾}{e}}_{2}=...={\underset{‾}{e}}_{m}$, there exists an i∈{1,2,...,m} such that $P_{i}\left({\underset{‾}{e}}_{i}\right)=0$. If not, $\forall i\;P_{i}\left({\underset{‾}{e}}_{i}\right)>0\;$. Further, any ${\underset{‾}{e}}_{i}$ can increase the utility of the equilibrium strategy by increasing ε, but this forms a contradiction. Thus, there must exist an i∈{1,2,...,m} where $P_{i}\left({\underset{‾}{e}}_{i}\right)=0$. Finally, according Lemma 2, ${\underset{‾}{e}}_{1}={\underset{‾}{e}}_{2}=...={\underset{‾}{e}}_{m}=0,$ which conflicts with ${\underset{‾}{e}}_{m}>{\underset{‾}{e}}_{m+1}=0.$

In the second situation, according to Lemma 2, obviously ${\underset{‾}{e}}_{2}=...={\underset{‾}{e}}_{n}=0$. If $P_{1}\left({\underset{‾}{e}}_{1}\right)$ = 0, $\delta ={\underset{‾}{e}}_{1}-{\underset{‾}{e}}_{2}$. If ${\underset{‾}{e}}_{1}$ decreases ε such that it falls into the interval $\left({\underset{‾}{e}}_{1},{\underset{‾}{e}}_{1}-\delta \right)$, obviously the utility function of $u_{i}\left({\underset{‾}{e}}_{i}-\varepsilon ,F_{-i}\right)=R-C\left(a_{i},{\underset{‾}{e}}_{i}-\varepsilon \right)$> $R-C\left(a_{i},{\underset{‾}{e}}_{i}\right)=\;u_{i}\left({\underset{‾}{e}}_{i},F_{-i}\right)$. This too contradicts the equilibrium strategy. Q.E.D. □

Lemma 4 $P_{1}\left(0\right)$ *= 0,* $P_{i}\left(0\right)$ *> 0,* ∀i∈{2,...,n}.

Proof:

According to Lemma 3, $\forall i,\;{\underset{‾}{e}}_{i}=0\;s$ince $C_{a_{1}}^{-1}\left(R\right)>C_{a_{2}}^{-1}\left(R\right)\geq {\bar{e}}_{i}\;\forall i\in \left\{2,...,n\right\}$. First, we can conclude $P_{1}\left(0\right)$ = 0 in the equilibrium strategy. Otherwise, if $P_{1}\left(0\right)>$ 0, it is a subsuperiority more than strategy that participant 1 bid 0 with a probability of 0 and bid $C_{a_{2}}^{-1}\left(R\right)$ with $P_{1}\left(0\right)$. This will increase the utility of participant 1 by at least $\left(C_{a_{1}}^{-1}\left(R\right)-C_{a_{2}}^{-1}\left(R\right)\right)\times P_{1}\left(0\right)$, which conflicts with the equilibrium strategy. The other obvious result is the equilibrium utility $u_{1}=\int u_{1}\left(e_{1},F_{-1}\right)dF_{1}\left(e_{1}\right)\geq \left(C_{a_{1}}^{-1}\left(R\right)-C_{a_{2}}^{-1}\left(R\right)\right)>0$.

Second, if there exists a j where $P_{j}\left(0\right)$ = 0, j∈{2,...,n}, i.e., $F_{j}\left(0\right)=0$. For any $\varepsilon \leq \;{\bar{e}}_{j}$, the utility of participant j falling within the interval [0,ε] is $\int_{0}^{\varepsilon }\left(R\prod_{i\neq j}F_{i}\left(x\right)-C\left(a_{j},x\right)\right)dF_{j}\left(x\right)$. Then, the utility of participant k≠j in the interval [0,ε] is $\int_{0}^{\varepsilon }\left(R\prod_{i\neq k}F_{i}\left(x\right)-C\left(a_{k},x\right)\right)dF_{k}\left(x\right)$ since$\lim_{x\to 0^{+}}R\prod_{i\neq k}F_{i}\left(x\right)=0$, $\lim_{x\to 0^{+}}C\left(a_{k},x\right)=0$. $F_{j}\left(x\right)$ in the equilibrium strategy is a cumulative distribution function, while $F_{j}\left(0\right)=0$. Participant j could therefore win against participant k by adjusting $F_{j}\left(x\right)$ in [0,ε] so as to make $R\prod_{i\neq k}F_{i}\left(x\right)-C\left(a_{1},x\right)<0$ in [0,ε]. In this situation, participant j increases his expected payoff by decreasing the expected payoff of others, which conflicts with the equilibrium strategy. To sum up, equilibrium strategy must satisfy that $P_{i}\left(0\right)$ > 0, ∀i∈{2,...,n}. Thus, each participant other than participant 1, who has the highest ability $a_{1}$, must put a mass point at 0. Q.E.D. □

Lemma 3 and Lemma 4 indicate that only participant 1will definitely take part in the competition.

The following section provides an analysis of the upper bound for ${\bar{e}}_{i}\;\forall i\in \left\{1,2,...,n\right\}$.

Lemma 5 $\;{\bar{e}}_{1}=\;{\bar{e}}_{2}=C_{a_{2}}^{-1}\left(R\right)$.

Proof:

Obviously $C_{a_{1}}^{-1}\left(R\right)>C_{a_{2}}^{-1}\left(R\right)>...>C_{a_{n}}^{-1}\left(R\right)$. According to Lemma 1, $C_{a_{2}}^{-1}\left(R\right)\geq {\bar{e}}_{2}$. First, ${\bar{e}}_{1}\geq \;{\bar{e}}_{i},\forall i\in \left\{2,...,n\right\}$ if ${\bar{e}}_{1}<{\bar{e}}_{j}\;for\;j\in \left\{2,...,n\right\}$. Participant 1 has at least a probability of $F_{j}\left({\bar{e}}_{j}\right)-F_{j}\left({\bar{e}}_{1}\right)$ where the utility≤0. If Participant 1 increases his effort to $\;C_{a_{2}}^{-1}\left(R\right)$. He can at least increase his utility by $\left(\;C_{a_{1}}^{-1}\left(R\right)-\;C_{a_{2}}^{-1}\left(R\right)\right)\times \left(F_{j}\left({\bar{e}}_{j}\right)-F_{j}\left({\bar{e}}_{1}\right)\right)$, which conflicts with the equilibrium strategy.

Second, it can be proven that ${\bar{e}}_{1}=C_{a_{2}}^{-1}\left(R\right)$. If ${\bar{e}}_{1}<C_{a_{2}}^{-1}\left(R\right)$, according to Lemma 4, then $P_{2}\left(0\right)$>0, which incentivizes Participant 2 to increase her effort from 0 to ${\bar{e}}_{1}$ to increase her payoff, which again conflicts with the equilibrium strategy. Thus, ${\bar{e}}_{1}=C_{a_{2}}^{-1}\left(R\right)$. If ${\bar{e}}_{2}\leq C_{a_{3}}^{-1}\left(R\right)<C_{a_{2}}^{-1}\left(R\right)$ because ${\bar{e}}_{i}<C_{a_{3}}^{-1}\left(R\right)\;\forall i\in \left\{3,4,...,n\right\}$. This leads to ${\bar{e}}_{1}\leq C_{a_{3}}^{-1}\left(R\right)\;$in equilibrium, which conflicts with ${\bar{e}}_{1}=C_{a_{2}}^{-1}\left(R\right)$. If $C_{a_{3}}^{-1}\left(R\right)<{\bar{e}}_{2}<C_{a_{2}}^{-1}\left(R\right)$, this obviously leads to ${\bar{e}}_{1}={\bar{e}}_{2}<C_{a_{2}}^{-1}\left(R\right)$, which also conflicts with ${\bar{e}}_{1}=C_{a_{2}}^{-1}\left(R\right).$ Q.E.D. □

Lemma 5 indicates the maximum effort of Participant 2 is the upper bound because Participant 1 exceeding the maximum effort of Participant 2 is an unnecessary loss and not an optimal solution for Participant 1.

Lemma 6 ${\bar{e}}_{i}=0\;for\;\forall i\in \left\{3,4,...,n\right\}$.

Proof:

Suppose that ${\bar{e}}_{j}=\max_{\forall i\in \{3,4,...,n\}}{\bar{e}}_{i}$. If ${\bar{e}}_{j}>0$, then $u_{j}\left({\bar{e}}_{j},F_{-j}\right)=R\prod_{i\neq j}F_{i}\left({\bar{e}}_{j}\right)-C\left(a_{j},{\bar{e}}_{j}\right)=0$. Otherwise, if $u_{j}\left({\bar{e}}_{j},F_{-j}\right)<0$, participant j is incentivized to exert zero effort instead of ${\bar{e}}_{j}$. If $u_{j}\left({\bar{e}}_{j},F_{-j}\right)>0$, this will incentivize participant j to exert ${\bar{e}}_{j}$ effort instead of none since, from Lemma 4, $P_{j}\left(0\right)>0$ and $u_{j}\left(0,F_{-j}\right)=0$, which conflicts with the equilibrium. Second, since $F_{2}\left(x\right)$ is an increasing function and $F_{2}\left({\bar{e}}_{2}\right)=1>F_{2}\left({\bar{e}}_{j}\right)$, $F_{j}\left({\bar{e}}_{j}\right)=1>F_{2}\left({\bar{e}}_{j}\right)$, then $R\prod_{i\neq 2}F_{i}\left({\bar{e}}_{j}\right)>R\prod_{i\neq j}F_{i}\left({\bar{e}}_{j}\right)$. Further, since C(a,x) is a decreasing function alongside ability a, $C\left(a_{j},{\bar{e}}_{j}\right)>C\left(a_{2},{\bar{e}}_{j}\right)$. Thus, the utility of Participant 2 exerting the effort ${\bar{e}}_{j}$ is $u_{2}\left({\bar{e}}_{j},F_{-2}\right)=R\prod_{i\neq 2}F_{i}\left({\bar{e}}_{j}\right)-C\left(a_{2},{\bar{e}}_{j}\right)>R\prod_{i\neq j}F_{i}\left({\bar{e}}_{j}\right)-C\left(a_{j},{\bar{e}}_{j}\right)=0$. And, from Lemma 4, $P_{2}\left(0\right)>0$, which incentivizes Participant 2 to exert ${\bar{e}}_{j}$ effort instead of none, in conflict to the equilibrium. Q.E.D. □

According to Lemma 6, it is obvious that $F_{i}\left(x\right)=1$ ∀i∈{3,4,...,n}. Based on these proofs, an open innovation contest with complete information will become a contest between two participants – Participants 1 and 2 – while the others in the contest will remain inactive. This means that only the two top participants will take part in the contest, while the others will withdraw. Here, the utility function can be formulated as a variational problem where

(9) $$\begin{array}{ccc} \max_{F_{1}}J\left(F_{1},F_{2}\right) & = & \int_{0}^{\;C_{a_{2}}^{-1}\left(R\right)}\left(R\times F_{2}\left(x\right)-C\left(a_{1},x\right)\right)F_{1}^{\prime }\left(x\right)dx\\ & & s.t.\;F_{1}\left(0\right)=0,F_{1}\left({\;C}_{a_{2}}^{-1}\left(R\right)\right)=1,{\;F}_{1}^{\prime }\left(x\right)>0 \end{array}$$

(10) $$\begin{array}{ccc} \max_{F_{2}}J\left(F_{1},F_{2}\right) & = & \int_{0}^{\;C_{a_{2}}^{-1}\left(R\right)}\left(R\times F_{1}\left(x\right)-C\left(a_{2},x\right)\right)F_{2}^{\prime }\left(x\right)dx\\ & & s.t.\;0<F_{2}\left(0\right)\langle 1,F_{2}\left(\;C_{a_{2}}^{-1}\left(R\right)\right)=1,F_{2}^{\prime }\left(x\right)\rangle 0 \end{array}$$

This leads to Proposition 1.

Proposition 1 *If* C(a,x) *has second-order differentiability in x, which means it has continuous first derivatives with* x*, a complete information contest will have a unique mixed-strategy equilibrium. That is, the solutions to* (9), (10)

(11) $$F_{1}\left(x\right)=\frac{C\left(a_{2},x\right)}{R}\;and\;\;F_{2}\left(x\right)=\frac{R-C\left(a_{1},x\right)+C\left(a_{1},\;C_{a_{2}}^{-1}\left(R\right)\right)}{R}$$

Proof:

The solutions to Eqs. (9) and (10) can be calculated in the space $C^{1}\left[0,\;C_{a_{2}}^{-1}\left(R\right)\right]$, which means the functions defined on $\left[0,\;C_{a_{2}}^{-1}\left(R\right)\right]$ will have continuous first derivatives. It can also be proven that the solution is a unique mixed-strategy equlibrium that satisfies Eqs. (9) and (10).

First, according to Theorem 1 of Chapter 1 in [33], the necessary condition for Eqs. (9) and (10) to have an extremum for $F_{1}\left(x\right)$ or $F_{2}\left(x\right)$ is to satisfy Euler's equation $\frac{d}{dx}G_{y^{\prime }}-G_{y}=0$, where $G=\left(R\times F_{2}\left(x\right)-C\left(a_{1},x\right)\right)F_{1}^{\prime }\left(x\right),\;y=F_{1}\left(x\right)$ for Eq. (9) and $G=\left(R\times F_{1}\left(x\right)-C\left(a_{2},x\right)\right)F_{2}^{\prime }\left(x\right),y=F_{2}\left(x\right)$ for Eq. (10). Solving Euler's equation for Eqs. (9) and (10), we have

(12) $$\begin{array}{ccc} \frac{d}{dx}G_{y^{\prime }}-G_{y} & = & \frac{d\left(R\times F_{2}\left(x\right)-C\left(a_{1},x\right)\right)}{dx}=0\\ & & R\times F_{2}\left(x\right)-C\left(a_{1},x\right)=C_{1},\;where\;C_{1}\;\text{is}\;a\;\text{constant} \end{array}$$

(13) $$\begin{array}{ccc} \frac{d}{dx}G_{y^{\prime }}-G_{y} & = & \frac{d\left(R\times F_{1}\left(x\right)-C\left(a_{2},x\right)\right)}{dx}=0\\ & & R\times F_{1}\left(x\right)-C\left(a_{2},x\right)=C_{2},\;\;where\;C_{2}\;\text{is}\;a\;\text{constant} \end{array}$$

Since $F_{2}\left(\;C_{a_{2}}^{-1}\left(R\right)\right)$ = 1, $C_{1}=R-C\left(a_{1},\;C_{a_{2}}^{-1}\left(R\right)\right)$ and $F_{2}\left(x\right)=\frac{R-C\left(a_{1},\;C_{a_{2}}^{-1}\left(R\right)\right)+C\left(a_{1},x\right)}{R}$. And, since $F_{1}\left(\;C_{a_{2}}^{-1}\left(R\right)\right)$ = 1, $C_{2}=0$ and $F_{1}\left(x\right)=\frac{C(a_{2},x)}{R}$. These are the solutions in Eq. (11), which also satisify the constraints in Eqs. (9) and (10). Also, due to the cost function, C(a,x) has a second derivative. Thus, $F_{1}\left(x\right)$ and $F_{2}\left(x\right)$ have continuous derivatives in that $C^{1}\left[0,\;C_{a_{2}}^{-1}\left(R\right)\right]$. Thus, $F_{1}\left(x\right)=\frac{C(a_{2},x)}{R}$, $F_{2}\left(x\right)=\frac{R-C\left(a_{1},\;C_{a_{2}}^{-1}\left(R\right)\right)+C\left(a_{1},x\right)}{R}$ is obviously the mixed-strategy equlibrium of Eqs. (9)and (10).

As previously calculated, $F_{1}\left(x\right)$ and $F_{2}\left(x\right)$ are a unique mixed-strategy equlibrium. If not, there must exist an $F_{1^{*}}\left(x\right)$ and an $F_{2^{*}}\left(x\right)$ that satisfy Eqs. (9) and (10) but differ from $F_{1}\left(x\right)$ and $F_{2}\left(x\right)$. Thus, there must exist an $x^{*}\in \left(0,\;C_{a_{2}}^{-1}\left(R\right)\right)$ such that $R\times F_{1^{*}}\left(x^{*}\right)-C\left(a_{2},x\right)>0$. Otherwise, for $\forall x\in (0,\;C_{a_{2}}^{-1}\left(R\right)),$ $R\times F_{1^{*}}\left(x\right)-C\left(a_{2},x\right)<0$. Thus, the optimal mixed-strategy is $F_{2^{*}}\left(x\right)=1$, i.e., not participating in the contest, which conflicts with Lemma 5. If $R\times F_{1^{*}}\left(x^{*}\right)-C\left(a_{2},x\right)>0$, according to Lemma 4, $P_{2}\left(0\right)$>0, Participant 2′s adjustment strategy is one of exerting the effort $x^{*}$ with $P_{2}\left(0\right)$ and no effort with 0. This increases his expected payoff, which conflicts with the mixed-strategy equlibrium. Q.E.D. □

**Fig. 1:**
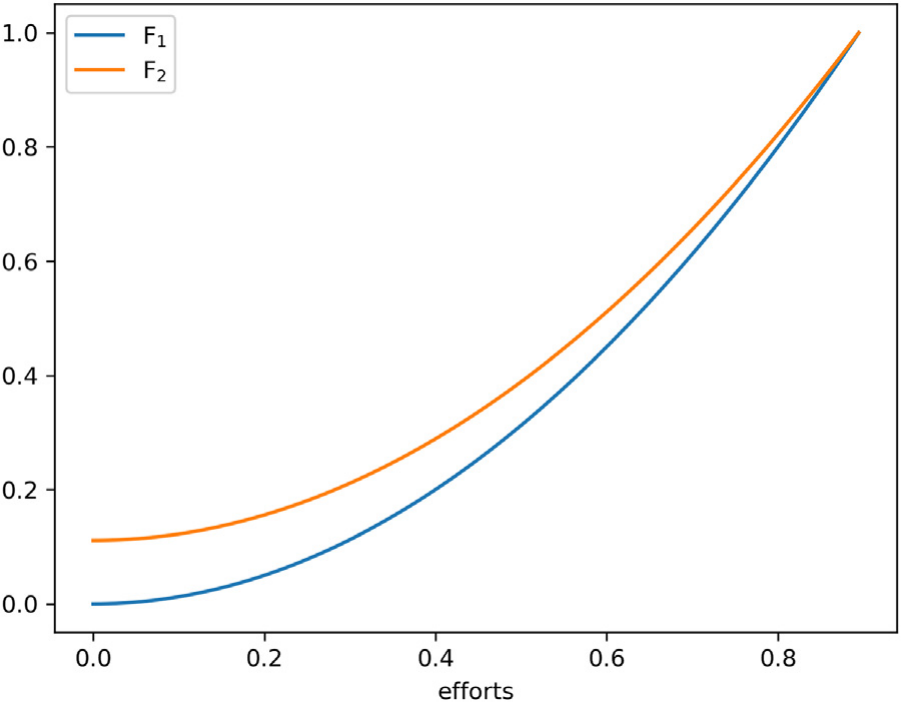
**The cumulative distribution functions**$F_{1}\left(x\right)$**and**$F_{2}\left(x\right)$**, when**$C\left(a,x\right)=\frac{x^{2}}{a}$**is defined as per [**16**,**^21^**], i.e.**,$\;a_{1}=0.9,$$a_{2}=0.8$**, and**R=1.

The above Proposition 1 reflects the fact that, in the competition, Participant 1′s strategy depends on Participant 2′s ability $a_{2}$, and vice versa. We can observe the shape of $F_{1}\left(x\right)$ and $F_{2}\left(x\right)$ in Fig. 1 when $C\left(a,x\right)=\frac{x^{2}}{a}$ is defined as per [16,21], and where$\;a_{1}=0.9,$ $a_{2}=0.8$, and R=1. Given Proposition 1 and Eq. (1), it is easy to reach Proposition 2.

Proposition 2 *If* C(a,x) *has continuous first derivatives with* x*, the sponsor's expected utility in a crowdsourcing context with complete information will be*

(14) $$\begin{array}{ccc} \Pi & = & E\left(\max_{i}U\left(q_{i}\right)\right)=E\left(max{\left(e_{1},e_{2}\right)}^{r}\right)\\ & = & \int_{0}^{\;C_{a_{2}}^{-1}\left(R\right)}max{\left(e_{1},e_{2}\right)}^{r}dx=\int_{0}^{\;C_{a_{2}}^{-1}\left(R\right)}\left(F_{1}\left(x\right)F_{2}^{\prime }\left(x\right)+F_{2}\left(x\right)F_{1}^{\prime }\left(x\right)\right)x^{r}dx\\ & = & \int_{0}^{\;C_{a_{2}}^{-1}\left(R\right)}\frac{x^{r}}{R^{2}}(C\left(a_{2},x\right)C_{x}^{\prime }\left(a_{1},x\right)\\ & & +C_{x}^{\prime }\left(a_{2},x\right)\left(C\left(a_{1},x\right)+R-C\left(a_{1},\;C_{a_{2}}^{-1}\left(R\right)\right)\right))dx \end{array}$$

Commonly, there are three types of cost functions depending on the type of task [20,21,27]. As an example, creative/innovation tasks have an increasing marginal cost, i.e., $C_{\mathrm{xx}}^{\prime \prime }\left(a_{1},x\right)>0$. The other two types have a constant or decreasing marginal cost, corresponding to $C_{\mathrm{xx}}^{\prime \prime }\left(a_{1},x\right)=0$ or $C_{\mathrm{xx}}^{\prime \prime }\left(a_{1},x\right)<0$. Such tasks might be coding jobs or similar. This research only focuses on analyzing the situations where the marginal costs are increasing as this is the most commonly encountered scenario in real-world applications [16,21], and particularly in open innovation contests.

### 3.2 When the marginal cost is increasing: $C\left(a,x\right)=\frac{x^{2}}{a}$

Using quantitative methods on qualitative research so as to compare the two mechanisms and observe their tendencies, the increasing marginal cost function $C\left(a,x\right)=\frac{x^{2}}{a}$ is defined as an incomplete information contest [16,21] that satisfies a decreasing function for a, and an increasing function for x, and where $C_{\mathrm{xx}}^{\prime \prime }\left(a_{1},x\right)>0$. From here, it is easy to arrive at $\;C_{a}^{-1}\left(R\right)=\sqrt{aR}$ and put it into Eq. (11). Thus, we have

(15) $$F_{1}\left(x\right)=\frac{C\left(a_{2},x\right)}{R}=\frac{x^{2}}{a_{2}R}$$

(16) $$F_{2}\left(x\right)=\frac{R-C\left(a_{1},\;C_{a_{2}}^{-1}\left(R\right)\right)+c\left(a_{1},x\right)}{R}=\frac{\left(a_{1}-a_{2}\right)R+x^{2}}{a_{1}R}$$

The expected effort expended by Participants 1and2 is therefore

(17) $$E\left(e_{1}\right)=\int_{0}^{\sqrt{a_{2}R}}xdF_{1}\left(x\right)=\frac{2\sqrt{a_{2}R}}{3}$$

(18) $$E\left(e_{2}\right)=\int_{0}^{\sqrt{a_{2}R}}xdF_{2}\left(x\right)=\frac{2a_{2}\sqrt{a_{2}R}}{3a_{1}}$$

According to Eq. (1), the sponsor's expected payoff $\Pi_{com}$ should be

(19) $$\begin{array}{ccc} \Pi_{com} & = & E\left(max{\left(q_{1},q_{2}\right)}^{r}\right)\\ & = & E\left(max{\left(e_{1},e_{2}\right)}^{r}\right)\\ & = & \frac{2a_{2}^{\frac{r}{2}}R^{\frac{r}{2}}}{2+r}+\left(\frac{4}{4+r}-\frac{2}{2+r}\right)\frac{a_{2}^{\frac{2+r}{2}}R^{\frac{r}{2}}}{a_{1}} \end{array}$$

Since $\frac{4}{4+r}-\frac{2}{2+r}>0$, when r>0, $\frac{\partial \Pi_{com}}{\partial a_{2}}>$ 0 and $\frac{\partial \Pi_{com}}{\partial a_{1}}<$ 0. Given a fixed $a_{1}$, $\Pi_{com}$ increases as $a_{2}$ increases, and given a fixed $a_{2}$, $\Pi_{com}$ increases as $a_{1}$ decreases, as Fig. 1 shows. However, $a_{1}$ is the upper bound of $a_{2}$, which suggests that the closer $a_{1}$ and $a_{2}$ are, the higher $\Pi_{com}$ will be, further proving the bonuses to be had from the competition.

Obviously, when r=1, the expected maximum bid for effort will be

$$E\left(max\left(e_{1},e_{2}\right)\right)=\frac{2a_{2}\sqrt{a_{2}R}}{15a_{1}}+\frac{2\sqrt{a_{2}R}}{3}$$

**Fig. 2:**
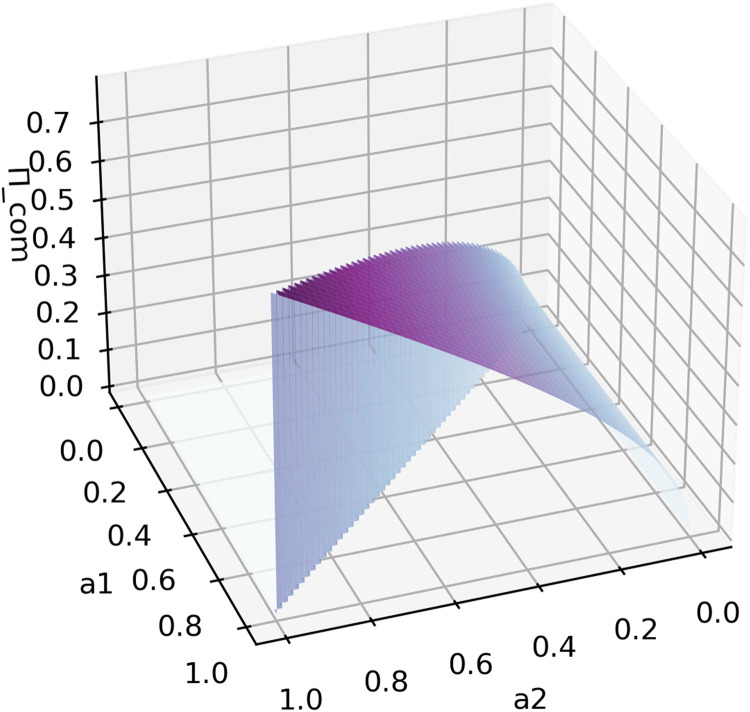
**Function image of**Eq. (19)**, the expected maximum sponsor's payoff as function of the maximum ability**$a_{1}$**and the second maximum ability**$a_{2}$**, with parameter**r=1**and**R=1.

Compared to the expected effort expended by Participant 1 in Eq. (15), it is easy to see that the quality gain for the sponsor from the competition will be $\frac{2a_{2}\sqrt{a_{2}R}}{15a_{1}}$. From Fig. 2, we can observe a competive phenomenon from how close abilities $a_{1}$ and $a_{2}$ are to each other.

In conclusion, the equilibrium strategy for a complete information contest sees the participants winnowed down to the top two players; the others will either remain inactive or quit the competition. The sponsor's expected suplus not only increases alongside the participants’ abilities, but also increases as the gap between the participants’ abilities decreases. This verifies the benefits to the sponsor from the participants competing. The next section offers a comparison between the results of this section and an incomplete information contest in terms of sponsor utility.

## 4 Comparison between complete and incomplete information contest

In a normal contest, one of the things a sponsor must decide is whether to have a registration phrase. Registration phases allow the sponsor to recruit participants, collect their information, and make a choice about whether to make the contestants’ individual abilities available to all participants after seeing the entrants. Without a registration phrase, the sponsor must decide whether to disclose the participants’ ability information in advance and without knowing who will be participating in the competition. This section provides some analyses from the sponsor's point of view as to whether ability information should be made available with and without a registration phase.

### 4.1 Comparison with a registration phase

When the sponsor has opted for a registration phase, he will have access to a general view of the participants’ profiles and can determine whether disclosing private information most benefits the contest based on the information provided. Thus, this discussion involves a comparison between which is better: making the information available or hiding it from the other competitors. Suppose a participant's abilities satisfy $1\geq a_{1}>a_{2}...>a_{n}>0$, where n is the total number of participants. Also assume that ability information is hidden from the participants. Thus, the participants’ beliefs about the abilities of others will follow a uniform distribution over the interval [0, 1], which is consistent with [21].

According to Proposition 2 in [21], in a winner-take-all contest with a given reward R, incomplete information, and an increasing marginal cost $C\left(a,x\right)=\frac{x^{2}}{a}$, the equilibrium strategy by participant i with ability $a_{i}$ for expending effort will be

(20) $$b_{incomplete}\left(a_{i}\right)=\sqrt{R\times \frac{\left(n-1\right)a_{i}^{n}}{n}},i=1,2,...,n.$$

And the sponsor's payoff $\Pi_{inc}$ will be

(21) $$\begin{array}{ccc} \Pi_{inc} & = & \max_{i}U\left(b_{incomplete}\left(a_{i}\right)\right)\\ & = & U\left(b_{incomplete}\left(a_{1}\right)\right)\\ & = & b_{incomplete}{\left(a_{1}\right)}^{r}\\ & = & {\left(R\times \frac{\left(n-1\right)a_{1}^{n}}{n}\right)}^{\frac{r}{2}} \end{array}$$

**Fig. 3:**
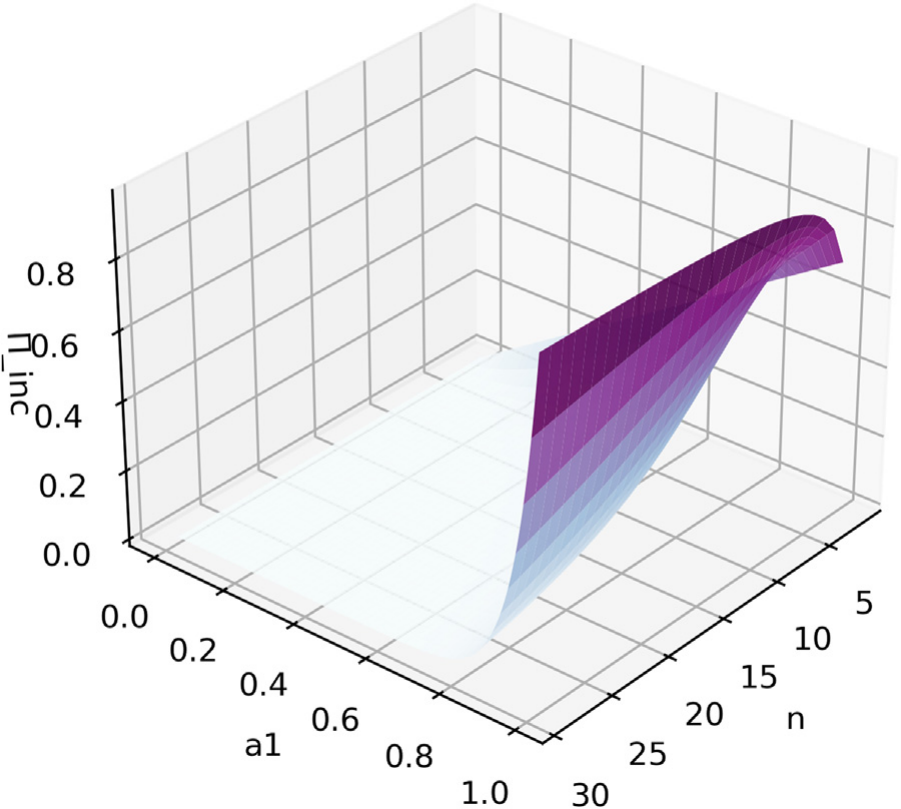
**Function image of**Eq. (21)**. The expected maximum sponsor's payoff as a function of the maximum ability of the participants**$a_{1}$**and the number of participants, where**r=1**and**R=1.

According to (21), $\Pi_{inc}$ will increase with an increase in $a_{1}$, but $a_{1}^{n}$ will decrease as n increases. Additionally, if $a_{1}\neq 1$, $\frac{\left(n-1\right)}{n}$ will increase as n increases. Thus, as shown in Fig. 3, $\Pi_{inc}$ has an inflection point as n increases, but will generally decrease as more participants enter the contest.

Corollary 1 *The necessary condition of a complete information equilibrium exceeds the incomplete information. Thus, the sponsor's payoff is*

(22) $$a_{2}>\frac{\left(n-1\right)a_{1}^{n}}{n}$$

Proof:

Obviously, the maximum effort given a mixed-strategy equilibrium and complete information must be more than the maximum effort given a pure strategy and incomplete information, i.e.,

(23) $$\begin{array}{ccc} & & \;C_{a_{2}}^{-1}\left(R\right)>\sqrt{R\times \frac{\left(n-1\right)a_{1}^{n}}{n}}\\ & & \;\sqrt{a_{2}R}>\sqrt{R\times \frac{\left(n-1\right)a_{1}^{n}}{n}}\\ & & \;a_{2}>\frac{\left(n-1\right)a_{1}^{n}}{n} \end{array}$$

Thus, Corollary 1 holds. Q.E.D. □

To compare a mixed-strategy equilibrium with complete information and a pure-strategy equilibrium, suppose $\Delta =\Pi_{com}-\Pi_{inc}\;$where the positive part of Δ is denoted as $\Delta^{+}$ so as to observe whether complete information is better.

(24) $$\Delta =\Pi_{com}-\Pi_{inc}=\frac{2a_{2}^{\frac{r}{2}}R^{\frac{r}{2}}}{2+r}+\left(\frac{4}{4+r}-\frac{2}{2+r}\right)\frac{a_{2}^{\frac{2+r}{2}}R^{\frac{r}{2}}}{a_{1}}-{\left(R\times \frac{\left(n-1\right)a_{1}^{n}}{n}\right)}^{\frac{r}{2}}$$

First, $\frac{\partial \Delta }{\partial a_{2}}=\frac{r}{2+r}a_{2}^{\frac{r-2}{2}}R^{\frac{r}{2}}+\left(\frac{4+2r}{4+r}-1\right)\frac{a_{2}^{\frac{r}{2}}R^{\frac{r}{2}}}{a_{1}}$ since r>0, $(\frac{4+2r}{4+r}-1)>0\;$and $\frac{\partial \Delta }{\partial a_{2}}>0$. Thus, Δ increases as $a_{2}$ increases. Additionally, $\frac{\partial \Delta }{\partial a_{1}}=\left(\frac{2}{2+r}-\frac{4}{4+r}\right)a_{2}^{\frac{2+r}{2}}R^{\frac{r}{2}}a_{1}^{-2}-R^{\frac{r}{2}}\frac{{\left(n-1\right)}^{\frac{r}{2}}rn}{2n^{\frac{r}{2}}}a_{1}^{\frac{rn-2}{2}}$ since r>0, $(\frac{2}{2+r}-\frac{4}{4+r})<0$ and $\frac{\partial \Delta }{\partial a_{1}}<0$. Thus, Δ decreases as $a_{1}$ increases.

**Fig. 4:**
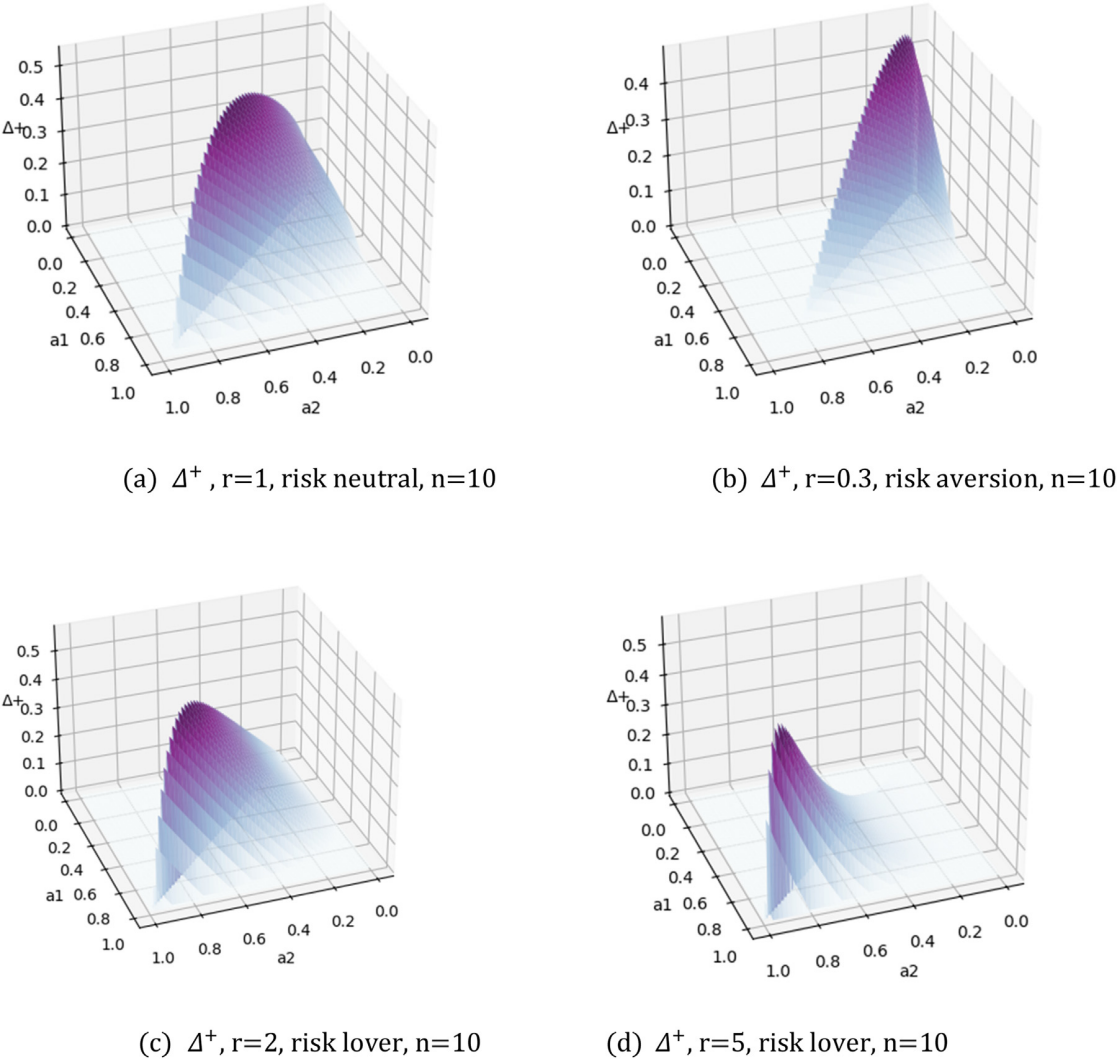
**Δ**^**+**^**from**Eq. (24)**with different values for**r.

Next, let us examine Eq. (24) with different risk preferences r. This involves simulating the positive part of the function Δ (denoted as $\Delta^{+}$) with different values for r, as shown in Fig. 4. In the figure, $\Delta^{+}$ represents the area in which complete information dominates incomplete information.

Fig. 4 shows the dominant areas move along $a_{1}=a_{2}$ to point (1,1) as r increases. This indicates that a risk-loving sponsor will benefit from a competition involving contestants of high ability. This can be interpreted as the possible distribution of effort exerted, which has an upper bound of$\;C_{a_{2}}^{-1}\left(R\right)$. This is more than the effort exerted in the incomplete information scenario. Further, observing the shape of Fig. 4, we can conclude that a risk-loving sponsor will benefit from the resulting fierce competition, given that $a_{1}$ and $a_{2}$ are close together.

In addition, since $a_{2}<a_{1}$, $\frac{\partial \Delta }{\partial a_{2}}>0$ and $\frac{\partial \Delta }{\partial a_{1}}<0$, it holds that the maximum of Δ is located on the line $a_{2}=a_{1}$, as also shown in Fig. 4. When $a_{2}=a_{1}$, $\Delta \left(a_{1}\right)=\frac{2a_{1}^{\frac{r}{2}}R^{\frac{r}{2}}}{2+r}+\left(\frac{4}{4+r}-\frac{2}{2+r}\right)a_{1}^{\frac{r}{2}}R^{\frac{r}{2}}-{\left(R\times \frac{\left(n-1\right)a_{1}^{n}}{n}\right)}^{\frac{r}{2}}$, $\frac{d\Delta (a_{1})}{da_{1}}=\frac{2R^{\frac{r}{2}}}{2+r}\frac{r}{2}a_{1}^{\frac{r-2}{2}}+\frac{r}{2}\left(\frac{4}{4+r}-\frac{2}{2+r}\right)a_{1}^{\frac{r-2}{2}}R^{\frac{r}{2}}-R^{\frac{r}{2}}\frac{{\left(n-1\right)}^{\frac{r}{2}}rn}{2n^{\frac{r}{2}}}a_{1}^{\frac{rn-2}{2}}$. And when $\frac{d\Delta (a_{1})}{da_{1}}=0$, we have $a_{1}^{\frac{r}{2}}\left(\frac{4}{4+r}-n{\left(\frac{n-1}{n}\right)}^{\frac{r}{2}}a_{1}^{\frac{(n-1)r}{2}}\right)=0$. Since $a_{1}>0$, the maximum point of $\Delta (a_{1})$ occurs when

(25) $$a_{1}={\left(\frac{\left(4+r\right)n^{\frac{r-2}{2}}}{4{\left(n-1\right)}^{\frac{r}{2}}}\right)}^{\frac{2}{\left(n-1\right)r}}$$

**Fig. 5:**
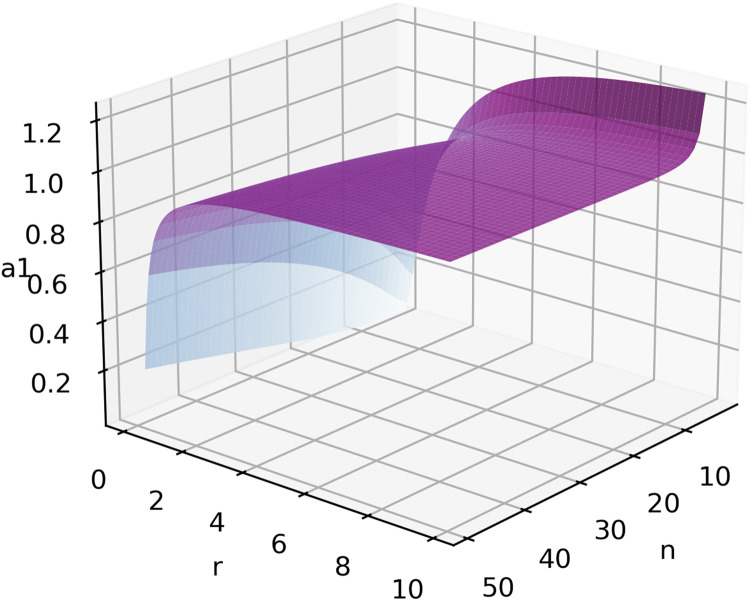
**Simulation of**Eq. (25)**, given 3 <*****n*****< 50 and 0.1 <*****r*****< 10**.

Fig. 5 simulates Eq. (25) with 2 < *n* < 50 and 0.1 < *r* < 10. Again, as $a_{1}$ increases, r increases. This result shows that the maximum of Δ moves to 1 as $a_{1}$ increases, which indicates that, given participants with strong abilities in the competition ($a_{1}$ and $a_{2}$) a risk-loving sponsor should prefer complete information.

However, taking a closer look at Fig. 4, one can see that the dominance area does not cover the region around point $a_{1}=a_{2}=1$, which means that, at this level, incomplete information will produce better outcomes than complete information, i.e., when $a_{1}=a_{2}\leq 1,\;\exists \varepsilon >0,\;$and $when\;1-\varepsilon <a_{1}=a_{2}\leq 1,\;\Delta >0.$ This indicates if the sponsor is able to attract the top participant in the field to the contest, an incomplete information competition is a better option.

In fact, when $a_{2}=a_{1}$, Eq. (24) can be rewritten as

(26) $$\begin{array}{ccc} \Delta {|}_{a_{2}=a_{1}} & = & \Pi_{com}-\Pi_{inc}=\frac{2a_{1}^{\frac{r}{2}}R^{\frac{r}{2}}}{2+r}+\left(\frac{4}{4+r}-\frac{2}{2+r}\right)\frac{a_{1}^{\frac{2+r}{2}}R^{\frac{r}{2}}}{a_{1}}\\ & & -\;{\left(R\times \frac{\left(n-1\right)a_{1}^{n}}{n}\right)}^{\frac{r}{2}}\\ & = & R^{\frac{r}{2}}a_{1}^{\frac{r}{2}}\left(\frac{4}{4+r}-{\left(\frac{\left(n-1\right)}{n}\right)}^{\frac{r}{2}}a_{1}^{\frac{\left(n-1\right)r}{2}}\right) \end{array}$$

Since $R>0\;and\;a_{1}>0,$ if <0, $\frac{4}{4+r}-{\left(\frac{\left(n-1\right)}{n}\right)}^{\frac{r}{2}}a_{1}^{\frac{(n-1)r}{2}}<0.$ Thus, an incomplete information contest will benefit the sponsor more than a complete information contest when

(27) $$a_{1}>{\left(\frac{4}{4+r}\right)}^{\frac{2}{\left(n-1\right)r}}{\left(\frac{n}{n-1}\right)}^{\frac{1}{n-1}}$$

**Fig. 6:**
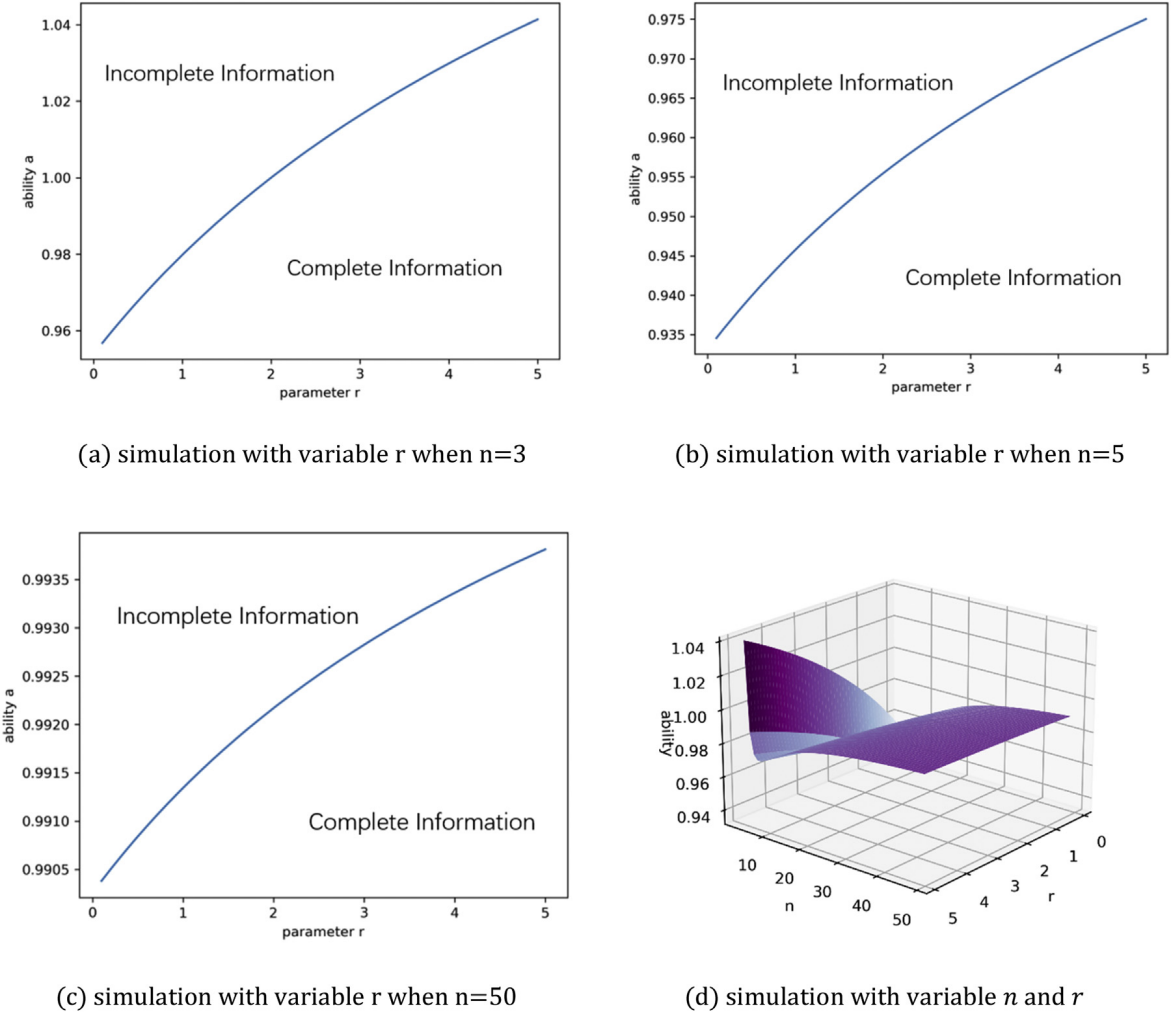
**Right side of (27)**.

In order to clearly observe the boundary between complete information and incomplete information, Fig. 6 presents a simulation of the right side of Eq. (27) when n=3,5, 50. The result is rather interesting. In the top half of the figure, the two panels show *n* = 3 and *n* = 5. Here, incomplete information has a probability of winning over complete information. Yet, when *n* = 50, the reverse is true as the bottom two panels show. Notably, this is not an intuitive conclusion because a complete information competition does not encourage the top players to expend more effort. However, with an increase in the risk parameter r, the ability curve increases. In other words, as the sponsor's preference for risk increases, complete information dominates an incomplete information contest. Fig. 6(d) reveals that, although the ability boundary between complete information and incomplete information decreases according to n first, it increases as n continuously increases, meaning that complete information dominates incomplete information when a large number of participants enter the contest.

### 4.2 Comparison without a registration phase

Without a registration phase, the sponsor must decide whether to make the participants’ ability information visible or invisible before any competitors even enter the contest. Unlike the previous model, with this model, the process by which the participants enter the contest needs to be simulated. The common assumption is that this is a sampling process in which the participants are independently drawn from same ability distribution via a cumulative distribution function Φ(x) defined in the range of [0,1]. Again, the total number of participants is denoted as n.

According to Proposition 1 in [27] and Proposition 2 in [21], the sponsor's payoff will increase depending on the maximum ability of the participants. More specifically, following Eq. (14) and [21,34], in an incomplete information contest, the sponsor's payoff will be associated with $a_{1}$, while in a complete information contest, the payoff will be associated with both $a_{1}$ and $a_{2}$. In terms of the process by which the participants enter the contest, the sponsor's payoff will depend on the order of statistics $X_{\left(1\right)}\;and\;X_{\left(2\right)}$, which are the first and second maximum ability values drawn from the same distribution via a cumulative distribution function Φ(x).

Suppose that there are n participants in the contest, with random abilities $X_{1},X_{2},...X_{n}\;$drawn independently using a cumulative distribution function Φ(x). Following [23,28], the maximum order statistics$\;a_{1}=$ $X_{\left(1\right)}$ will have a cumulative distribution function of

(28) $$\begin{array}{ccc} F_{a_{1}}\left(x\right) & = & Pr\left(X_{\left(1\right)}\leq x\right)\\ & = & Pr\left(X_{1}\leq x\right)\;Pr\left(X_{2}\leq x\right)\ldots Pr\left(X_{n}\leq x\right)\\ & = & {\left[Pr\left(X_{1}\leq x\right)\right]}^{n}={\left[\Phi \left(x\right)\right]}^{n} \end{array}$$

According to Eq. (14), the expected payoff given complete information will depend on the joint distribution of $a_{1}$ and $a_{2}$, which is the joint distribution of $X_{\left(1\right)}=a_{1}$ and $X_{\left(2\right)}=a_{2}$ with a cumulative distribution function of

(29) $$\begin{array}{ccc} & & F_{a_{1},a_{2}}\left(x,y\right)=Pr\left(X_{\left(1\right)}\leq x,X_{\left(2\right)}\leq y\right)\\ & & \;=\left\{ \begin{array}{cc} Pr\left(X_{\left(1\right)}\leq x\right) & x<y\\ Pr\left(X_{\left(1\right)}\leq y,X_{\left(2\right)}\leq y\right)+Pr\left(y<X_{\left(1\right)}\leq x,X_{\left(2\right)}\leq y\right) & x\geq y \end{array}\right. \end{array}$$

It is evident that, if x<y, $F_{a_{1},a_{2}}\left(x,y\right)=\;Pr\left(X_{\left(1\right)}\leq x\right)=F_{a_{1}}\left(x\right)={\left[\Phi (x)\right]}^{n}\;$. If x≥y, the first term of Eq. (29) $Pr\left(X_{\left(1\right)}\leq y,X_{\left(2\right)}\leq y\right)=Pr\left(X_{\left(1\right)}\leq y\right)=F_{a_{1}}\left(y\right)={\left[\Phi (y)\right]}^{n}$. For the second term,

(30) $$\begin{array}{ccc} & & Pr\left(y\leq X_{\left(1\right)}\leq x,X_{\left(2\right)}\leq y\right)\\ & & \;=\sum_{i=1}^{n}Pr\left(y\leq X_{i}\leq x,X_{j\neq i}\leq y\right)\\ & & \;=\sum_{i=1}^{n}Pr\left(y\leq X_{i}\leq x\right)\prod_{j\neq i}Pr\left(X_{j}\leq y\right)\\ & & \;=nPr\left(y\leq X_{1}\leq x\right)Pr\left(X_{2}\leq y\right)\ldots Pr\left(X_{n}\leq y\right)\\ & & \;=n\left[\Phi \left(x\right)-\Phi \left(y\right)\right]{\left[\Phi \left(y\right)\right]}^{n-1} \end{array}$$

To sum up, the cumulative distribution function of the joint distribution for $X_{\left(1\right)}$ and $X_{\left(2\right)}$ is

(31) $$F_{a_{1},a_{2}}\left(x,y\right)=\left\{ \begin{array}{cc} {\left[\Phi \left(x\right)\right]}^{n} & if\;x<y\;\\ {\left[\Phi \left(y\right)\right]}^{n}+n\left[\Phi \left(x\right)-\Phi \left(y\right)\right]{\left[\Phi \left(y\right)\right]}^{n-1} & if\;x\geq y \end{array}\right.$$

#### 4.2.1 The sponsor's expected payoff with an increasing marginal cost $C\left(a,x\right)=\frac{x^{2}}{a}\;$and uniform ability distribution Φ(x)=U(0,1)

This section adopts the same hypotheses as [16,21] where $C\left(a,x\right)=\frac{x^{2}}{a}$ and Φ(x)=U(0,1). According to Eqs. (14) and (18), the sponsor's expected payoff with complete information is

(32) $$\begin{array}{ccc} \Pi_{com} & = & E_{a_{1},a_{2},\ldots ,a_{n}∼\Phi \left(x\right)}\left(E\left(\max_{i}U\left(q_{i}\right)\right)\right)\\ & = & E_{a_{1},a_{2},\ldots ,a_{n}∼\Phi \left(x\right)}\left(E\left(max{\left(e_{1},e_{2}\right)}^{r}\right)\right)\\ & = & \int \int_{0}^{\;C_{a_{2}}^{-1}\left(R\right)}max{\left(e_{1},e_{2}\right)}^{r}dxdF_{a_{1},a_{2}}\\ & = & \int \frac{2a_{2}^{\frac{r}{2}}R^{\frac{r}{2}}}{2+r}+\left(\frac{4}{4+r}-\frac{2}{2+r}\right)\frac{a_{2}^{\frac{2+r}{2}}R^{\frac{r}{2}}}{a_{1}}dF_{a_{1},a_{2}}\\ & = & \int_{0}^{1}dx\int_{0}^{x}\left(\frac{2y^{\frac{r}{2}}R^{\frac{r}{2}}}{2+r}+\left(\frac{4}{4+r}-\frac{2}{2+r}\right)\frac{y^{\frac{2+r}{2}}R^{\frac{r}{2}}}{x}\right)\\ & & \times \left(n\left(n-1\right)y^{n-2}\right)dy\\ & = & \frac{8n\left(n-1\right)R^{\frac{r}{2}}}{\left(2+r\right)\left(r+2n-2\right)\left(r+2n\right)}+\frac{4n\left(n-1\right)R^{\frac{r}{2}}}{{\left(r+2n\right)}^{2}}\left(\frac{4}{4+r}-\frac{2}{2+r}\right) \end{array}$$

**Fig. 7:**
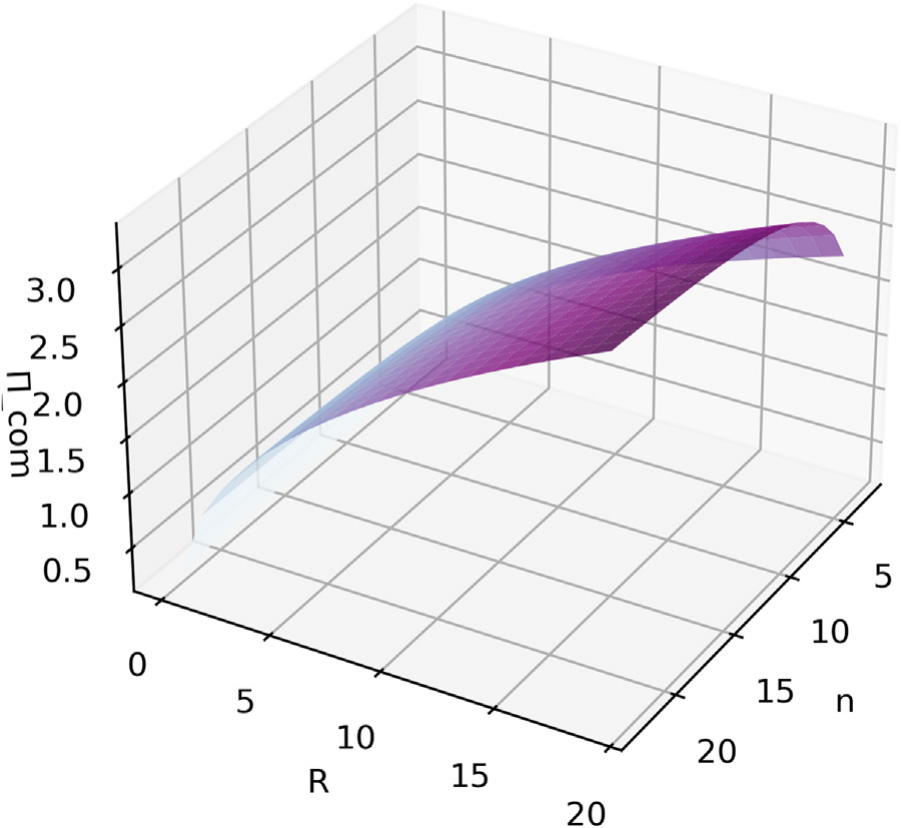
**simulation of**$\Pi_{com}$**with variable**n**and**Rwhenr=1.

Let us set r, the risk parameter, to r=1. And since ${\frac{\partial \Pi_{com}}{\partial n}|}_{r=1}=\frac{28n^{4}+4n^{3}-n^{2}+4n+1}{{\left(8n^{3}-4n^{2}-2n-1\right)}^{2}}\times \frac{32R^{\frac{r}{2}}}{15}>0$ and ${\frac{\partial \Pi_{com}}{\partial R}|}_{r=1}=\left(\frac{8n(n-1)}{3(2n-1)(2n+1)}+\frac{8n(n-1)}{15{\left(2n+1\right)}^{2}}\right)\frac{1}{2R^{\frac{1}{2}}}>0$ when n≥andR>0, it indicates that the sponsor will benefit from there being more entrants in the competition and also from offering higher rewards. Fig. 7 shows $\Pi_{com}$ with different values for n and R. Here, the limitation of Eq. (32) tends to $\lim_{n\to +\infty }\Pi_{com}=\frac{4R^{\frac{r}{2}}}{4+r}$. From this, one can draw the conclusion that R determines the upper limit of the payoff, while an increase in n increases the probability of approaching the upper limit.

Given incomplete information as per Proposition 2 in [21], in any winner-take-all contest and given any reward R with incomplete information and an increasing marginal cost $C\left(a,x\right)=\frac{x^{2}}{a}$, the equilibrium strategy of participant i with ability $a_{i}$ in terms of expending effort will be the same as in Eq. (20). And the sponsor's payoff without an intermediary fee will be

(33) $$\begin{array}{ccc} \Pi_{inc} & = & E_{x_{i}∼U\left(0,1\right)}\left(\Pi \right)\\ & = & E_{x_{i}∼U\left(0,1\right)}\left(\max_{i}U\left(q_{i}\right)\right)\\ & = & \;E_{x_{i}∼U\left(0,1\right)}\left(b_{incomplete}{\left(a_{1}\right)}^{r}\right)\\ & = & \int_{0}^{1}\sqrt{{\left(R\times \frac{\left(n-1\right)x_{\left(1\right)}^{n}}{n}\right)}^{r}}dF_{x_{\left(1\right)}∼{\left[U\left(0,1\right)\right]}^{n}}\left(x\right)\\ & = & \frac{2R^{\frac{r}{2}}}{r+2}{\left(\frac{n-1}{n}\right)}^{\frac{r}{2}} \end{array}$$

**Fig. 8:**
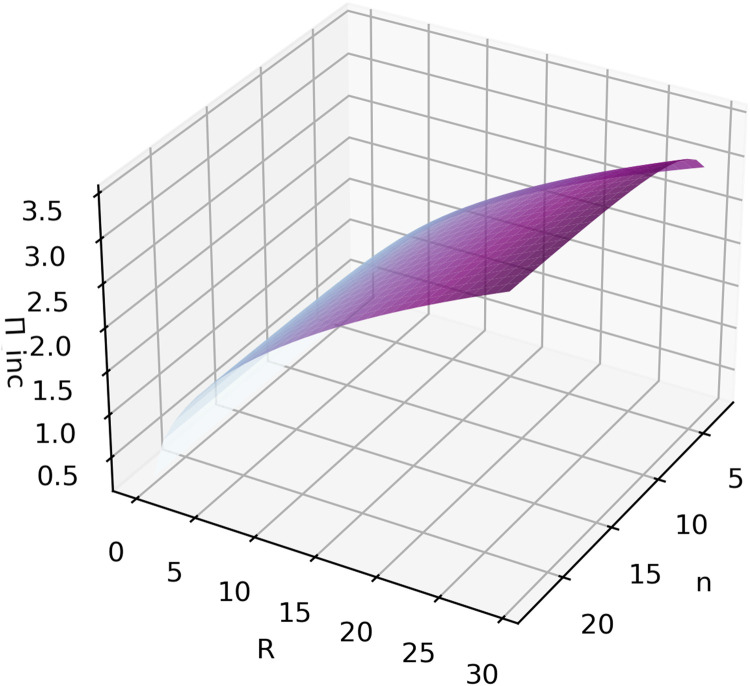
**Simulation of**$\Pi_{inc}$**with the variables**n**and**R**when*****r*****=1**.

Setting the risk parameter r again to 1 in the complete information scenario, ${\frac{\partial \Pi_{inc}}{\partial n}|}_{r=1}>0$ and ${\frac{\partial \Pi_{inc}}{\partial R}|}_{r=1}>0$, which shows that the sponsor again benefits from more entrants and that higher rewards will incentivize more effort from the top participants. Fig. 8 shows $\Pi_{inc}$ with different values for n and R. Further, the limitation of Eq. (33) will tend to $\lim_{n\to +\infty }\Pi_{inc}=\frac{2R^{\frac{r}{2}}}{r+2}$. This leads to the same conclusion that the upper limit of the sponsor's payoff is determined by reward the R, and that an increase in n increases the probability of approaching this limit.

#### 4.2.2 Comparison of the complete and incomplete information scenarios

Using the same approach as for Eq. (24) to observe the difference in equilibriums between the complete and incomplete information scenarios, Δ is defined as $\Delta =\Pi_{com}-\Pi_{inc}\;$and used to observe whether complete information is better.

(34) $$\begin{array}{ccc} \Delta & = & \Pi_{com}-\Pi_{inc}\\ & = & \frac{8n\left(n-1\right)R^{\frac{r}{2}}}{\left(2+r\right)\left(r+2n-2\right)\left(r+2n\right)}+\frac{4n\left(n-1\right)R^{\frac{r}{2}}}{{\left(r+2n\right)}^{2}}\left(\frac{4}{4+r}-\frac{2}{2+r}\right)-\frac{2R^{\frac{r}{2}}}{r+2}{\left(\frac{n-1}{n}\right)}^{\frac{r}{2}} \end{array}$$

**Fig. 9:**
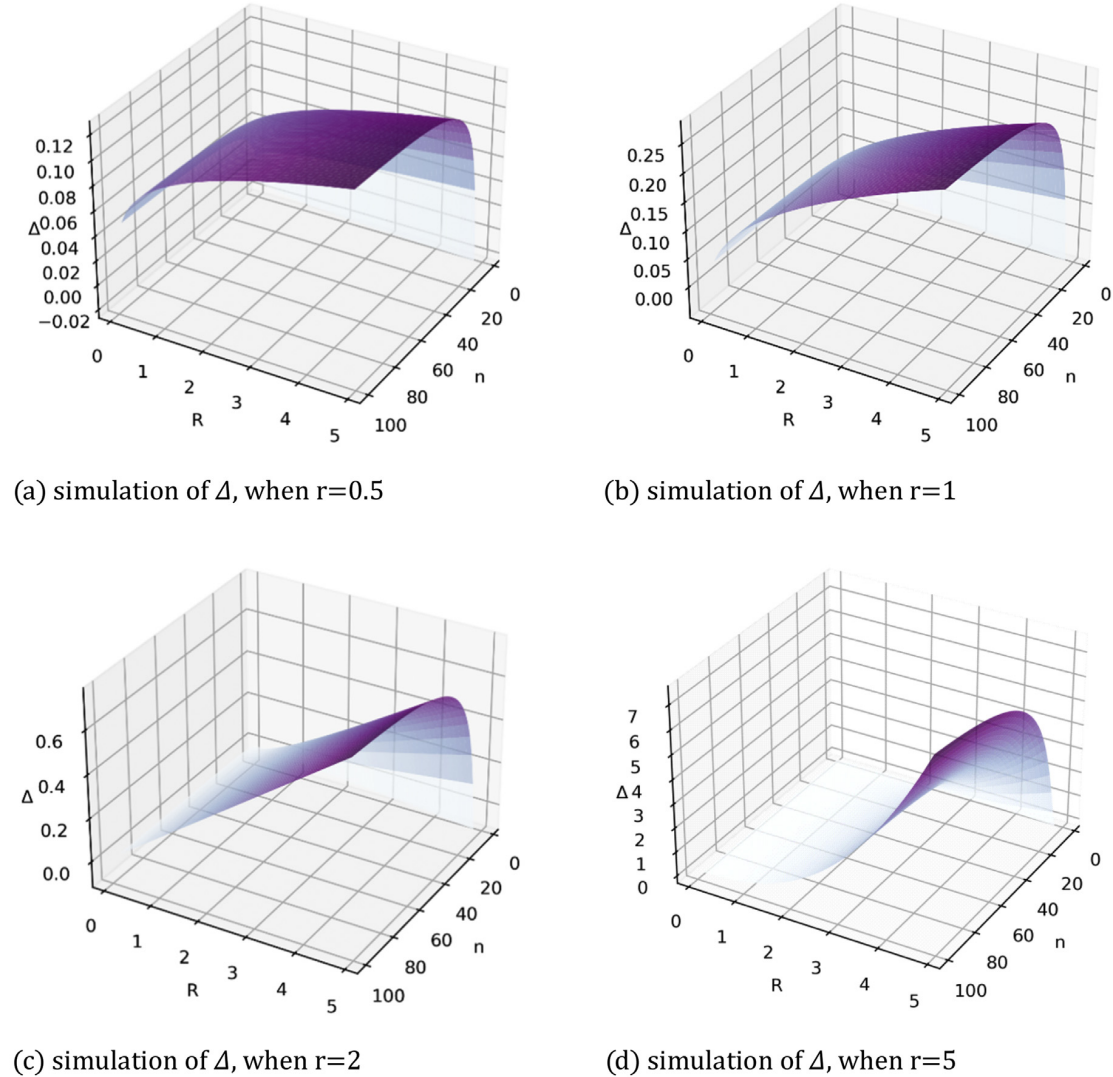
**Simulation of**Δ**in (35) with variable**n, R**and r**.

Obviously, $\lim_{n\to +\infty }\Delta ={\text{lim}}_{n\to +\infty }\Pi_{com}-{\text{lim}}_{n\to +\infty }\Pi_{inc}=\frac{4R^{\frac{r}{2}}}{4+r}-\frac{2R^{\frac{r}{2}}}{r+2}=\frac{2rR^{\frac{r}{2}}}{\left(r+2)(r+4\right)}$. Since r>0, ${\text{lim}}_{n\to +\infty }\;\Delta >0$, which indicates that when the number of participants grows large enough, complete information is better than incomplete information in a contest without a registration phase. Fig. 9 shows Δ with different values for n, R, and r. As shown by the four surfaces, complete information always yields a better payoff than incomplete information. Further, Δ increases with n and R, while R deters the upper limit, approaching it with n. Additionally, the variations in the surfaces with different values for r demonstrate that a sponsor's risk preference amplifies Δ, i.e., that a risk-taking sponsor will benefit more from visible ability information when there is no registration phase.

## 5 Discussion and implications

Overall, these analyses show that, in most cases, a complete information contest is better for the sponsor when the participants have strong abilities in the problem domain. The exception is when the contest has a registration phase and a participant's ability exceeds a certain threshold, as shown in Fig. 4. In this case, incomplete information is better than complete information. Without a registration phase, complete information will always yield more of a payoff than incomplete information, and this payoff will increase depending on the level of risk the sponsor is willing to take. Thus, for risk-loving sponsor, making the names and abilities of the competitors available to all the participants is the preferred choice. That said, the upper limit of the payoff is determined by the reward R, making the reward the decisive factor in the contest's return for the sponsor. The above conclusion is based upon the assumption that the participants’ abilities are drawn from a random distribution. However, if the sponsor is able to invite the top expert in the field to join the contest, they should probably opt for an incomplete information contest and conduct the competition like it is a closed tender.

This research reveals the important role information disclosure can play when holding single-stage open innovation contests. The theoretical proofs provided characterize mixed-strategy eqillibriums between contestants in complete information scenarios, reflecting that competition tends to boil down to a contest between the top two most able players. As such, complete information will generally persuade contestants beyond the top two to withdraw. This is confirmed by two popular buzzwords in the Chinese online media ‘Tang-Ping’ or ‘lying flat’ [35], which refers to the phenomenon of withdrawing from a contest [35,36], and ‘Nei Juan’ or ’involution’ meaning ‘excessive competition’ [36], being the reason why competitors withdraw [36,37]. Many researchers have studied these concepts from the perspective of cultural value [35], social pressures [36,38], and decreased economic impetus [38]. However, no one has yet interpreted these phenomena from the standpoint of a microeconomic behavior model. From this analysis, I find that ‘lying flat’ refers to the inactive participants revealed in Lemma 6 and its complete information model, while ‘involution’ is the increasing of number of participants n. Under the hypothesis that the participants’ abilities are drawn from same distribution via a cumulative distribution function Φ(x), for participant i≤n, the probability that her ability $a_{i}\;\text{ranks}\;\text{out}\;\text{of}\;\text{top}\;\text{two},\;\text{such}\;\text{that}\;\Phi \left(a_{i}\right)<1,$ is

(36) $$\begin{array}{ccc} P\left(a_{i}\leq X_{\left(2\right)}\right) & = & 1-P\left(a_{i}\geq X_{\left(2\right)}\right)\\ & = & 1-\;P\left(a_{i}=X_{\left(2\right)}\right)-P\left(a_{i}=X_{\left(1\right)}\right)\\ & = & 1-\Phi {\left(a_{i}\right)}^{n-1}-\left(n-1\right)\left(1-\Phi \left(a_{i}\right)\right)\Phi {\left(a_{i}\right)}^{n-2} \end{array}$$

Obviously, Eq. (36) will tend to 1 when n→+∞. This demonstrates why ’involution’ leads to ‘lying flat’. However, an effective solution for ‘lying flat’ is to keep the contestants’ ability information private. Let us assume that this social competition spans more than a single-stage. From Eq. (20), which is a monotonic invertible function for a∈[0,1], after one-stage of an incomplete information competition, the participants will be able to deduce the quality of their opponents’ abilities. Hence, in the following stage, the competition becomes a complete information contest. Consequently, from a long-term, multi-round perspective, crowdsourcing competitions are generally a game of complete information.

## 6 Conclusion

In most studies of winner-take-all contest models, the researchers only focus on incomplete information [16,19,21,27]. This study, however, considers both incomplete and complete information contests, drawing comparisons between the two in competitions both with and without a registration phase. With a registration phase, the sponsor can collect information about the participants before he decides whether to make that information available to all contestants. Without a registration phrase, the sponsor must decide ‘blind’, that is without knowing who will ultimately participate. The difference between the two scenarios is the sampling process by which participants enter the competition, Further, so as to compare a mixed-strategy equilibrium with complete information and pure-strategy equilibrium with incomplete information, a risk parameter is included in the sponsor's utility function, which models his risk preference. This is because a mixed-strategy approach will always lead to greater expected payoff. Moreover, we numerically simulated the equilibrium strategy and created a surface graph for better insight.

The main results are as follows. The first analysis concerns a complete information contest formulated as a variational problem. In the mixed-strategy equilibrium, only the top two players exerted effort, the others chose to remain inactive. The second analysis compares the registration/no registration scenarios. In the registration scenario, the result was counter-intuitive in that when a player's ability exceeded a certain threshold, an incomplete information competition prompted the top player to expend more effort than in a complete information competition, even when the top two players had abilities of a similar strength. Without a registration phase, a complete information contest always yielded better results than an incomplete information contest. Third, we used the mixed-strategy equilibrium in a complete information contest to interpret an economic phenomenon known as ‘involution’ and ‘lying flat’ that has recently received a great deal of attention in China. It refers to players withdrawing from a contest due to excessive competition. Previous researchers have focused on psychological explanations for this phenomenon. This study is the first attempt to explain the behavior as an equilibrium strategy.

Overall, this research reveals the importance of information disclosure when holding single-stage open innovation contests. In the future, we will conduct empirical validations by surveying contest participants and analyzing the competition results provided by sponsors. Modelling multiple-price and muliple-stage complete information contests and comparing the results with incomplete information contests is also left to future work, as are qualitative analyses with more realistic assumptions, such as a non-uniform distribution of participant abilities.

## Declaration of competing interest

The authors declare that they have no conflicts of interest in this work.
